# Rare Presentation of Pulmonary Alveolar Proteinosis Causing Acute Respiratory Failure

**DOI:** 10.1155/2016/4064539

**Published:** 2016-05-16

**Authors:** Ryan R. Kroll, Sameer Kumar, Ronald F. Grossman, Charles Price, John R. Srigley

**Affiliations:** ^1^Department of Internal Medicine, Queen's University, Etherington Hall, Room 3033, 94 Stuart Street, Kingston, ON, Canada K7L 3N6; ^2^Trillium Health Partners-Credit Valley Hospital Site, Mississauga, ON, Canada L5M 2N1; ^3^University of Toronto, Trillium Health Partners-Credit Valley Hospital Site, Mississauga, ON, Canada L5M 2N1; ^4^Laboratory Medicine and Genetics Program, Trillium Health Partners-Credit Valley Hospital Site, Mississauga, ON, Canada L5M 2N1

## Abstract

Pulmonary alveolar proteinosis (PAP) is a rare condition characterized by dysfunctional alveolar macrophages, which ineffectively clear surfactant and typically cause mild hypoxemia. Characteristic Computed Tomography findings are septal reticulations superimposed on ground-glass opacities in a crazy paving pattern, with a clear juxtaposition between affected and unaffected parenchyma. While traditionally PAP was diagnosed via biopsy, bronchoalveolar lavage (BAL) is usually sufficient; the fluid appears milky, and on microscopic examination there are foamy macrophages with eosinophilic granules and extracellular hyaline material that is Periodic Acid-Schiff positive. Standard therapy is whole lung lavage (WLL), although novel treatments are under development. The case presented is a 55-year-old woman with six months of progressive dyspnea, who developed hypoxemic respiratory failure requiring mechanical ventilation; she had typical findings of PAP on imaging and BAL. WLL was ultimately successful in restoring adequate oxygenation. Respiratory failure of this magnitude is a rare finding in PAP.

## 1. Learning Themes

- Pulmonary alveolar proteinosis is an uncommon disease that typically presents with mild illness. In rare instances, it can progress acutely to hypoxemic respiratory failure.
- While there are new treatments in development, whole lung lavage remains the standard of care for treating pulmonary alveolar proteinosis.

## 2. Pretest

- What are characteristic findings of pulmonary alveolar proteinosis on Computed Tomography?
- How is the diagnosis of pulmonary alveolar proteinosis made in most cases?

## 3. Case Presentation

A 55-year-old woman presented to an Ontario emergency department with six months of progressive exertional dyspnea, associated with a cough productive of a scant amount of grey sputum. The dyspnea had become so severe that even hair brushing elucidated symptoms. There were no infectious or cardiac symptoms. The patient described a ten-pound weight loss but no other constitutional symptoms. There was no exposure to radiation, chemotherapy, dusts, solvents, smoke, livestock, or other causes of interstitial lung disease. There was no exposure to sick contacts or tuberculosis and no features of connective tissue disease. The patient had no allergies but was a current smoker of 25 pack-years. Her medical history included depression and a nephrectomy for donation. She took tiotropium and budesonide/formoterol at home but had no documented lung disease. The patient's other medications were doxepin and pantoprazole.

On examination, she was afebrile, with a heart rate of 92 beats per minute, a respiratory rate of 40 breaths per minute, an oxygen saturation of 86% on room-air (improving to 95% on 50% oxygen by facemask), and a blood pressure of 127/87 millimeters of mercury. Physical examination revealed coarse bibasilar crackles and digital clubbing. The remainder of the examination was noncontributory. The patient was admitted to Internal Medicine for further investigation and management.

**Figure 1:**
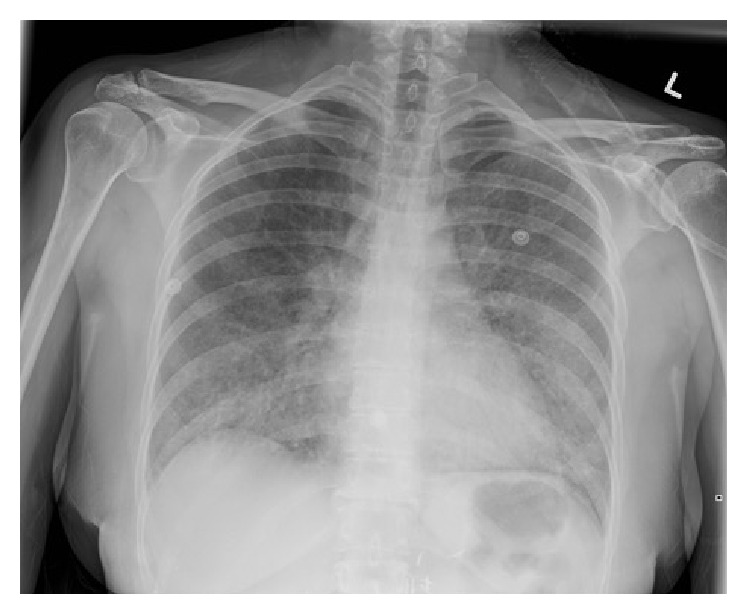
Initial chest radiograph, posterior-anterior view, showing hazy airspace opacification of the lower lobes bilaterally.

**Figure 2:**
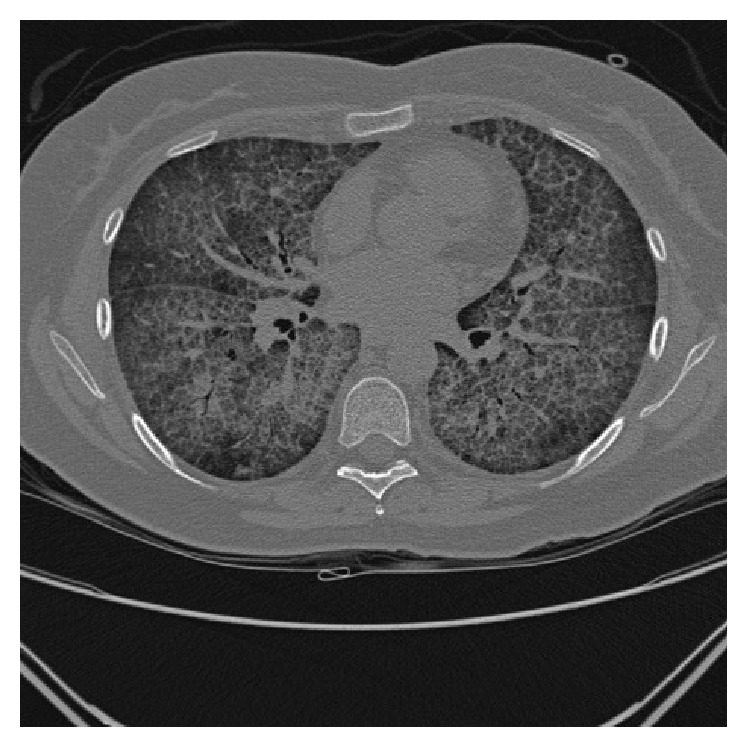
CT of the thorax depicting diffuse bilateral ground-glass opacities with intralobular septal thickening; the findings are reported by the radiologist to be a crazy paving pattern.

Laboratory studies demonstrated a hemoglobin of 161 grams (g)/litre (L) (normal range 115–155 g/L), while platelets and leukocytes, including differential, were unremarkable. Her electrolytes were normal with the exception of potassium at 5.1 millimoles (mmol)/L (normal range 3.5–5 mmol/L). Initial chest radiograph demonstrated hazy airspace opacification of the lower lobes bilaterally (Figure 1). Computed Tomography (CT) of the thorax revealed diffuse bilateral ground-glass opacities with intralobular septal thickening, as well as mild mediastinal, pretracheal, and para-aortic lymphadenopathy (Figure 2). The findings were reported as a crazy paving pattern.

**Figure 3:**
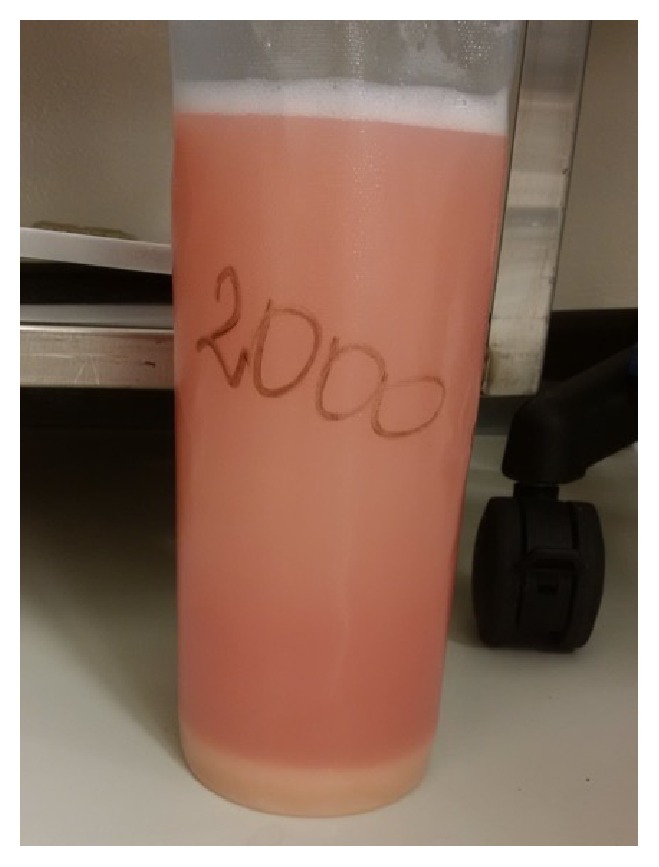
Photograph of the BAL fluid, demonstrating an opaque, milky appearance.

**Figure 4:**
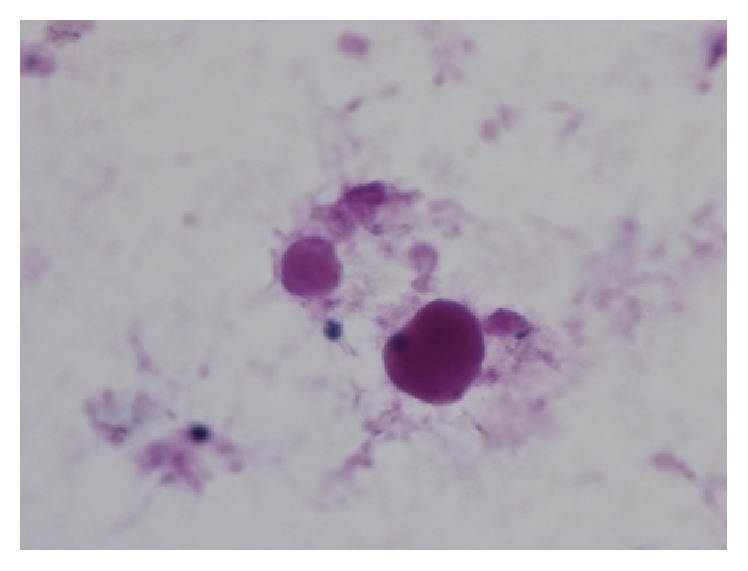
PAS-stained BAL preparation showing PAS positive globular material and supporting a diagnosis of PAP (magnification 400x).

**Figure 5:**
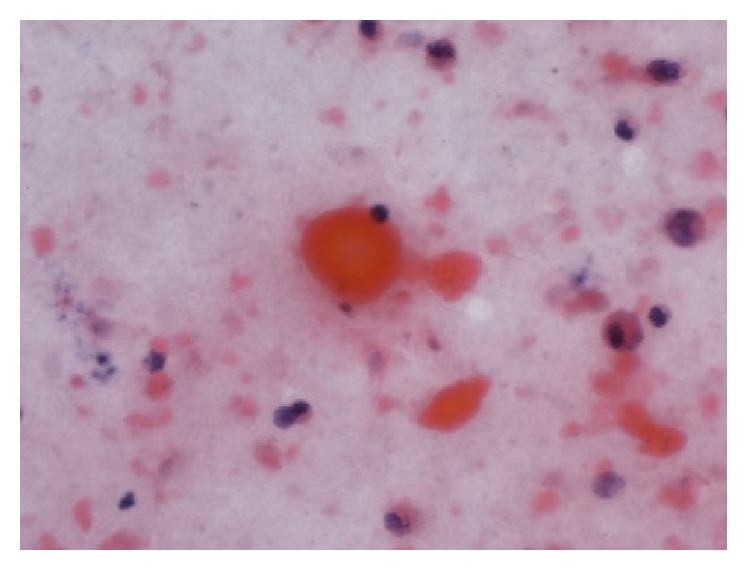
Papanikolaou stained BAL preparation showing abundant globular orangeophilic material along with a few macrophages and leukocytes (magnification 400x).

On postadmission day two the patient became increasingly hypoxemic and required intubation. She was transferred to the Intensive Care Unit and underwent bronchoscopy with bronchoalveolar lavage (BAL). BAL fluid from all lobes appeared thin and milky (Figure 3). Under microscopic examination, the fluid contained scattered globular Periodic Acid-Schiff positive material, with no malignant cells, fungi, or hemosiderin-laden macrophages (Figures 4 and 5). BAL cultures were negative for microorganisms and contained abundant debris. The laboratory was unable to perform a cell count on the sample. BAL findings and CT results were sufficient for diagnosis of pulmonary alveolar proteinosis (PAP); this was presumed to be autoimmune given the lack of potential secondary causes. It remains unclear what may have contributed to the clinical severity.

**Figure 6:**
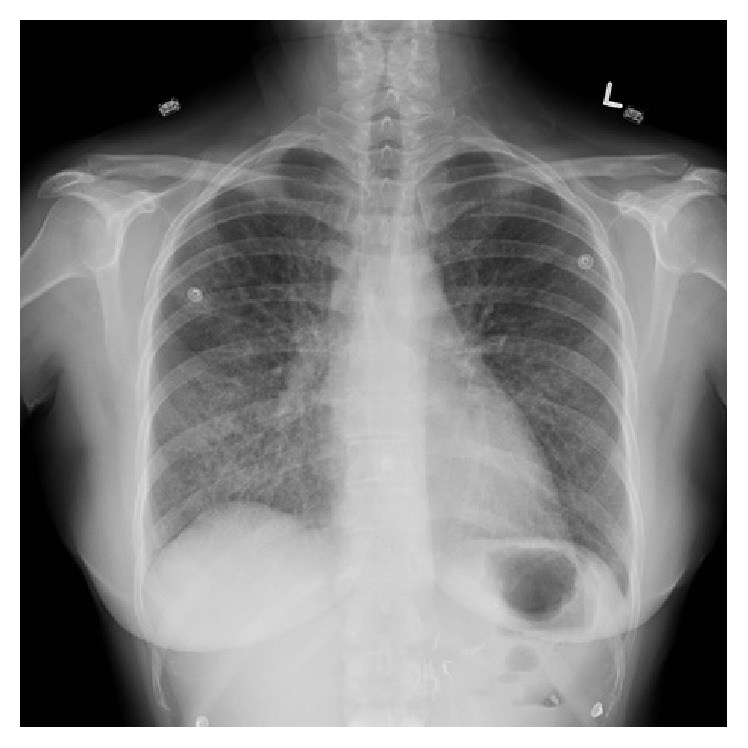
Posterior-anterior chest radiograph after three sessions of WLL, demonstrating improvement of the infiltrative process.

Three sessions of whole lung lavage (WLL) over a one-week period improved the patient's oxygenation sufficiently for extubation. A radiograph taken before discharge showed improvement in her airspace disease (Figure 6). She was discharged home six days after extubation with Respirology follow-up.

## 4. Discussion

Pulmonary alveolar proteinosis, first described in 1958, is a rare condition with an estimated prevalence of 3.7–6.2 cases per million people, usually affecting the middle-aged, with a 2 : 1 predilection for males [1, 2]. The disease manifests as an inability to clear alveolar surfactant due to macrophage dysfunction, leading to hypoxemia [1]. PAP can be divided into three entities: hereditary, secondary, and autoimmune [1]. Hereditary PAP is associated with mutations in the Granulocyte-Macrophage Colony-Stimulating Factor (GM-CSF) receptor leading to signaling abnormalities and macrophage dysfunction [1]. In secondary PAP, macrophage dysfunction is thought to be associated with hematologic malignancies, immunodeficiency, and exposure to organic and inorganic dusts [1, 2]. Autoimmune PAP comprises the majority of cases [1]. These individuals can have Immunoglobulin G (IgG) anti-GM-CSF antibodies, which bind to GM-CSF, inhibiting its ability to bind to GM-CSF receptors, thus causing macrophage dysfunction [1, 3].

The diagnosis of PAP can be challenging. While one-third of patients are asymptomatic, patients often present with progressive exertional dyspnea and cough [2, 3]. Less commonly, patients present with chest discomfort, weight loss, fatigue, fever, and small-volume hemoptysis [3]. It is a rare but recognized complication for patients to develop respiratory failure and require mechanical ventilation [4–8]. Physical examination is often unremarkable, although clubbing is present in 30% of patients, and fine crackles may be present [2, 3]. While no diagnostic biomarker exists, IgG anti-GM-CSF autoantibodies may be useful in diagnosing autoimmune PAP [2].

Imaging is crucial in diagnosing PAP. Chest radiography is nonspecific but classically demonstrates bilateral alveolar opacities most visible in the perihilar region, resembling pulmonary edema or opportunistic infection [2, 3]. CT is more specific in detecting PAP; findings suspicious for PAP are septal reticulations superimposed on ground-glass opacities, referred to as crazy paving [3]. There is usually a clear juxtaposition between affected and unaffected parenchyma in a geographic distribution [2, 3]. The differential diagnosis for crazy paving is broad and encompasses a multitude of pulmonary conditions (Table 1) [9]. Large focal consolidation is a rare finding which suggests opportunistic infection [3]. Pulmonary function testing typically demonstrates a restrictive defect, decreased total lung and vital capacity, hypoxemia with a raised alveolar-arterial gradient, and a disproportionately reduced diffusion capacity of carbon monoxide [1, 2].

Traditionally, lung biopsy was required for diagnosing PAP; however advancements in bronchoalveolar lavage (BAL) have made more invasive sampling unnecessary in most cases [2, 3]. The BAL fluid in PAP is typically milky in appearance but can be variable depending on whether it originated in affected or unaffected parenchyma [3]. Proteinaceous material collects in alveoli, terminal bronchioles, and macrophages, with scattered cholesterol clefts and type II pneumocyte hyperplasia [1]. Cytological examination usually demonstrates increased cellularity (330,000 cells/milliliter) and an increased proportion of lymphocytes [3]. Few large, foamy macrophages with eosinophilic granules are present, with extracellular globular hyaline material that is homogenously Periodic Acid-Schiff positive and Alcian Blue stain negative [3]. Alveolar architecture is usually well-preserved in PAP [3]. Electron microscopy of the BAL fluid reveals tubular myelin, lamellar bodies, and fused membrane structures which are morphologically identical to surfactant [2, 3].

Treatment of hereditary PAP is not well-characterized [3]. In secondary PAP, management includes treating the associated condition. In all forms of PAP, including autoimmune PAP, the removal of proteinaceous material is accomplished via WLL [3, 8, 10]. Technological advancements have allowed for the use of general anesthesia and extracorporeal membrane oxygenation in performing WLL in more difficult cases [11]. While WLL techniques vary, no randomized control trials have demonstrated superiority of any one technique [10]. WLL is generally well-tolerated and is effective in two-thirds of patients, although multiple treatments are sometimes required [8, 11]. An emerging treatment is the use of subcutaneous and inhaled GM-CSF, which has been shown in several smaller studies to be effective as an adjunct or as monotherapy [3, 11]. Although rituximab and plasmapheresis show promise, they are not considered standard therapy [11].

The prognosis of PAP varies depending on etiology; five-year survival of autoimmune PAP approaches 95% with optimal therapy [3]. While this is not the first PAP case report in* Canadian Respiratory Journal*, the severity contrasts with the case published in 2012 by Patel et al. [10]. This degree of respiratory compromise is highly atypical of PAP and highlights the variability in presentation of a rare disease. This case serves as a reminder to clinicians to maintain a broad differential diagnosis in acutely ill patients; clinicians should consider PAP when crazy paving is identified, to facilitate early intervention.

## 5. Posttest

- What are characteristic findings of pulmonary alveolar proteinosis on CT?
   Typical CT findings are septal reticulations superimposed on ground-glass opacities, referred to as a crazy paving pattern. There is typically a clear juxtaposition between affected and unaffected parenchyma in a geographic distribution.
- How is the diagnosis of pulmonary alveolar proteinosis made in most cases?
   Bronchoalveolar lavage is sufficient for diagnosis in most cases, without the need for a biopsy.

## Figures and Tables

**Table 1:** Differential diagnosis of crazy paving on CT imaging, adapted from De Wever et al. [9].

Acute diseases	Subacute/chronic diseases
Pulmonary edema	Usual interstitial pneumonia
Infection (viral, bacterial, *Pneumocystis jirovecii*, and mycoplasma)	Nonspecific interstitial pneumonia
Pulmonary hemorrhage	Pulmonary alveolar proteinosis
Acute interstitial pneumonia	Organizing pneumonia
Acute respiratory distress syndrome	Vasculitis (Churg-Strauss syndrome)
Radiation pneumonitis	Eosinophilic pneumonia (chronic)
Eosinophilic pneumonia	Malignancy
	Lymphangitic spread of malignancy
	Sarcoidosis
	Lipoid pneumonia
	Alveolar microlithiasis
	Barium aspiration
